# IL-6 Induction by TNFα and IL-1β in an Osteoblast-Like Cell Line

**Published:** 2010-06

**Authors:** Elena Confalone, Giuseppe D’Alessio, Adriana Furia

**Affiliations:** *Department of Structural and Functional Biology University Federico II of Naples, Campus of Monte S. Angelo, via Cinthia, Naples, Italy*

**Keywords:** stress cytokines, antioxidant, osteosarcoma cells

## Abstract

Stress cytokines tumor necrosis factor α, interleukin-1 β and interleukin-6 modulate the activity of a variety of cell types including osteoblasts, and are involved in the pathogenesis of several rheumatic diseases associated with systemic bone loss. We have studied the expression of interleukin-6 induced by interleukin-1 β and tumor necrosis factor α in the osteoblast-like cell line MG-63, derived from a human osteosarcoma. We have observed marked differences in the regulation of interleukin-6 gene expression by tumor necrosis factor α or interleukin-1 β, at the level of mRNA steady state and stability and cytokine secretion. In addition, N-acetyl cysteine, a scavenger of reactive oxygen species, inhibits activation of NF-κB and induction of interleukin-6 by tumor necrosis factor α, being ineffective on interleukin-1 β activity. These data illustrate the action of stress cytokines on a cell line widely used in *in vitro* studies as a reliable model of osteoblast response to cytokines involved in bone resorbing diseases, an important issue for developing new strategies for treatments of bone diseases.

## INTRODUCTION

Tumor necrosis factor α (TNFα) and interleukin-1 β (IL-1β) are multifunctional cytokines which mediate various inflammation and cellular immune responses (1, 2). Their actions are in part due to the ability to regulate the expression of other genes *via* activation of transcription factors, among which nuclear factor-κB (NF-κB) is known to play a pivotal role in the regulation of pro-inflammatory genes. NF-κB is a ubiquitous, heterodimeric transcription factor of the REL family, located in the cytoplasm as an inhibited complex. Upon stimulation of cells with inducers, NF-κB is activated by phosphorylation, polyubiquitination and proteasomal degradation of the κB inhibitory moiety (IκB) (3), leading to nuclear translocation of NF-κB and transcriptional activation of the responsive genes. Signaling required for NF-κB activation by TNFα (4) or IL-1β (5) has been largely elucidated. These cytokines activate a distinct class of receptor molecules and signal trasducers, which finally form oligomers including TRAF proteins in both pathways. TRAF2 in TNFα pathway and TRAF6 in IL-1β pathway function as ubiquitin ligases promoting the synthesis of lysine 63-linked polyubiquitin chains. Ubiquitination is required for activation of tumor growth factor β activated kinase 1 (6), which in turn is responsible for phosphorylation and activation of IκB kinases. These enzymes specifically phosphorylate the regulatory adjacent Ser residues located in the N-terminal region of IκB (7), leading to its proteasomal degradation.

Reactive oxygen species (ROS) also play a role in TNFα and IL-1β signaling, although the mechanisms by which the cognate receptors activate ROS production are not completely understood. It has been reported that in a number of cell types, activation of the small GTPase Rac1 and NADPH oxidase is involved in this process. Spontaneous or enzyme-catalyzed dismutation of O_2_^•-^ produces H_2_O_2_ which is thought to direct redox-dependent recruitment of TRAFs and activation of NF-κB (8, 9). However, it has been shown that that the requirement and the biochemical source of ROS in these pathways are cell type specific (10).

A number of genes have been shown to be transcriptionally regulated by NF-κB, among them the gene encoding interleukin-6 (IL-6), another stress cytokine. IL-6 plays a central role in homeostasis of the immune system, in modulation of acute-phase protein synthesis in hepatocytes and in physiopathological bone resorption by osteoclasts. TNFα and IL-1β activate IL-6 synthesis in osteoblasts and this cytokine is known to modulate both osteoblast and osteoclast differentiation, playing an important role in the pathogenesis of diseases associated with systemic bone loss and subchondral bone erosions (11, 12). TNFα and IL-1β are the most potent inducers of IL-6 gene expression, which is mainly regulated at the transcriptional level. It has been clearly established that NF-κB plays the main role in this regulation (13), *via* a κB site at positions -73 to -64 located within the promoter region of the IL-6 gene.

In this study we have compared the activity of TNF and IL-1 on the MG-63 osteoblastoma cells, as a model for cytokine regulated IL-6 expression. Any difference in the ability to regulate IL-6 gene activation is indeed an important issue for the development of treatment strategies in bone disease.

## MATERIALS

Cell culture medium RPMI and fetal calf serum were from Gibco BR, IL-1β and TNFα were purchased from Genzyme. SP6 RNA polymerase was from Promega, RNase A, *Aspergillus oryzae* RNase T1 and proteinase K were purchased from Worthington, Sequenase from USB. Oligonucleotides were from Genset. The Biotrack human IL-6 ELISA system and α^32^ PdCTP were from Amersham, Kodak films were used for autoradiography. All other chemicals were from SIGMA.

## METHODS

MG-63 osteosarcoma cells were cultured in RPMI, with L-glutamine, gentamycin (50 μg/ml) and 10% fetal calf serum, in 5% CO_2_ at 37°C. N-acetyl cysteine (NAC), was added to cell cultures as indicated in the text. Cell viability was evaluated with the Trypan blue method. Cells were stimulated with 20 ng of IL-1β or TNFα, producing maximal response of IL-6 secretion. Actynomycin D was used at the concentration of 25 μg/ml for up to 48 hours. In all experimental conditions tested cell viability was at least 95%.

IL-6 in conditioned medium was quantified with the Biotrack human IL-6 ELISA system, according to the manifacturer’s instructions. Optical density at 450 nm was determined on a plate reader.

RNA was purified from cell cultures by acid phenol extraction and RNA integrity was checked in denaturing agarose gel. For RNase protection assay, the antisense RNA probe was obtained by synthesis on the pGEM-1ghIL-6, kindly provided by Dr Haegman, Ghent University. This plasmid contains 1200 bp of the 5’ flanking sequence and part of the transcribed sequence of the IL-6 gene. The template plasmid was linearized with PvuII at a site located within the second intron, then added to a reaction mixture containing dATP, dUTP, dGTP, 2.5 μCi/μl of α^32^ PdCTP (10 mCi/ml) and 1 U/μl of SP6 RNA polymerase. After 1 hour incubation at 37°C, DNAase was added and incubated for additional 15 min The probe, spanning exons 1 and 2, the first intron and part of the second one of the IL-6 gene, was purified with phenol-chloroform extraction, precipitated in ethanol and dissolved in a buffer containing 40 mM PIPES pH6.7, 1 mM EDTA, 0.4 M NaCl and 80% formamide. 15 μg of total RNA were then annealed with an excess of the radiolabelled RNA probe (10^9^ cpm/μg), in a buffer containing PIPES pH 6.7, 1 mM EDTA, 0.4 M NaCl and 80% formamide, for 18 hours at 42°C. The mixture was digested for 1 hour at 37°C with bovine pancreatic RNase A and *Aspergillus oryzae* RNase T1, then with proteinase K, extracted twice with phenol-chloroform and precipitated with ethanol. The size of the protected probe, corresponding to exon 2 of the IL-6 mRNA, was 191 nucleotides. The probe for β-actin mRNA was prepared with a similar procedure, using as a template a plasmid containing the human β-actin coding sequence. The resulting antisense transcript was added to each reaction mixture as an internal standard and produced a protected fragment of 84 nt. Samples were then dissolved in PIPES-formamide buffer and analysed in 6% denaturing polyacrylamide gel in 0.5×TBE, run at 250 V then dried and exposed for autoradiography. Densitometric analysis was performed with UltroScan XL-LKB.

Nuclear extracts were prepared as already described (14). Protein concentration was measured by the Bradford method (Bio-Rad). In EMSA assay, 5 μg of nuclear extract were incubated with the double stranded radiolabeled probe (5’-ATGTGGGATTTTCCCCTG-3’, ~0.01 pmol; specific activity 1-2 × 10^6^ c.p.m./pmol) added last to each reaction mixture in 20 μl of buffer (50 mM Tris/Cl, 0.1 M NaCl, 0.1 μg of poly(dI-dC)·poly(dI-dC), 1 mM DTT, 1% NP-40, 4% glycerol) and incubated at room temperature for 20 min. The oligonucleotide probe was designed with three 5’ protruding nucleotides on both ends for labelling with Sequenase, α-^32^P dATP and α-^32^P dCTP. Unincorporated nucleotides were removed by Sephadex G-50 column chromatography. In competition assays, a 100-fold molar excess of unlabeled double-stranded probe was added to the reaction mixture. Samples were loaded on a 5% native polyacrylamide gel in 0.5×TBE, run at 250 V, dried and analyzed by desitometry as described above.

## RESULTS

### Time-course of IL-6 mRNA induction

**Figure 1:**
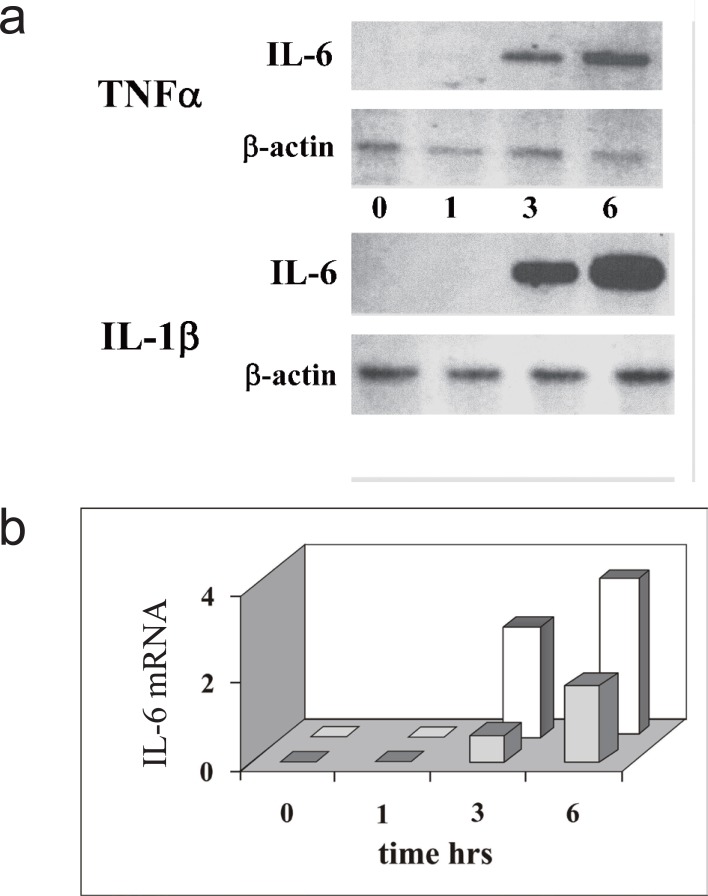
IL-6 mRNA induction triggered by TNFα or IL-1β in MG-63 ostesarcoma cells. a, RNase protection assay of IL-6 mRNA. The panel show the fragment protected by IL-6 mRNA and the fragment protected by the β-actin mRNA, as an internal standard. Cells were harvested and RNA purified at 0, 1, 3 or 6 hours after cytokine addition; b, Densitometric analysis of IL-6 mRNA (arbitrary unit) induced by cell treatment with TNFα (grey) or IL-1β (white), the amount of IL-6 mRNA was normalized versus β-actin mRNA. The data shown in the figure are representative of two independent experiments.

In many cell types, IL-6 induction by TNFα or IL-1β is mainly regulated at the transcriptional level leading to the accumulation of the specific transcript. Thus, to compare the action of these cytokines in the osteosarcoma cell line MG-63, we have first monitored the profile of IL-6 mRNA accumulation as a function of time, measuring the steady-state level of the transcript. RNA was purified from MG-63 cells before or after 1, 3 or 6 hours of cytokine treatment, then analyzed by RNase protection assay using an antisense RNA fragment spanning exons 1 and 2, the first intron and part of the second exon of the human IL-6 gene. The basal level of the specific mRNA is negligible in these cells, but it can be clearly detected after 3 hr of cytokine treatment (Fig. 1a), either with TNFα or IL-1β. The amount of the message accumulated up to six hours is higher with IL-1β than with TNFα stimulation, as shown by densitometric analysis of the IL6 mRNA specific signal relative to that of β-actin mRNA (Fig. 1b). Nuclear NF-κB, the main transcription factor in IL-6 gene activation, is already detectable in nuclear extracts 30 min after cytokine treatment, preceding the appearance of IL-6 mRNA and is still active up to six hours (compare lanes 1-4 and 9, 10 of Fig. 4 and Fig. 5b).

### Stability of IL-6 mRNA

**Figure 2:**
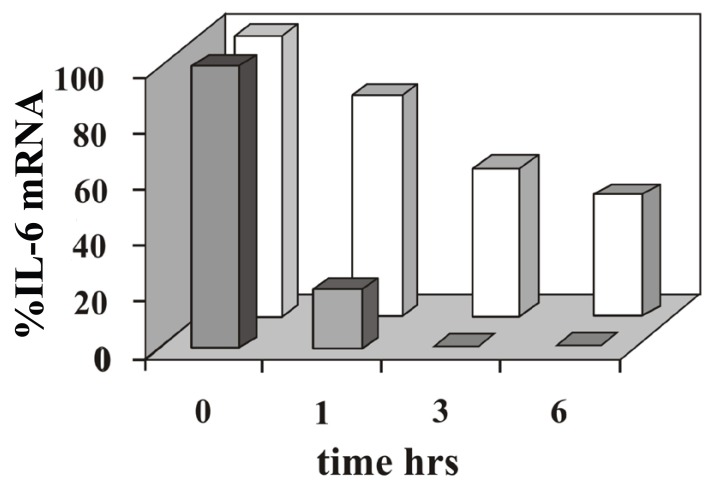
Analysis of IL-6 mRNA decay in cells treated with TNFα or IL-1β. Cells treated for 4 hours with TNFα or IL-1β were cultured in the presence of actinomycin D and harvested at the indicated time. Total RNA samples were analysed by RNase protection assay. In densitometric analyses the amount of IL-6 mRNA induced by TNFa (grey) or IL-1β (white), was normalized versus β-actin mRNA. The data shown in the figure are representative of two independent experiments.

We asked if post-transcriptional regulation might play a role in the difference observed in the IL-6 mRNA expression induced by TNFα or IL-1β. To clarify this, we carried out a time course RNase protection analysis of total RNA purified from MG-63 cells stimulated with the cytokines in the presence of actinomycin D, to inhibit *the novo* transcription. The result of this experiment, shown in Fig. 2, clearly indicate that the half-life of IL-6 mRNA is shorter in cells stimulated with TNFα, thus the higher amount of IL-6 mRNA accumulated upon IL-1β treatment is in part due to the higher stability of the transcript.

### IL-6 secreted in conditioned medium

**Figure 3:**
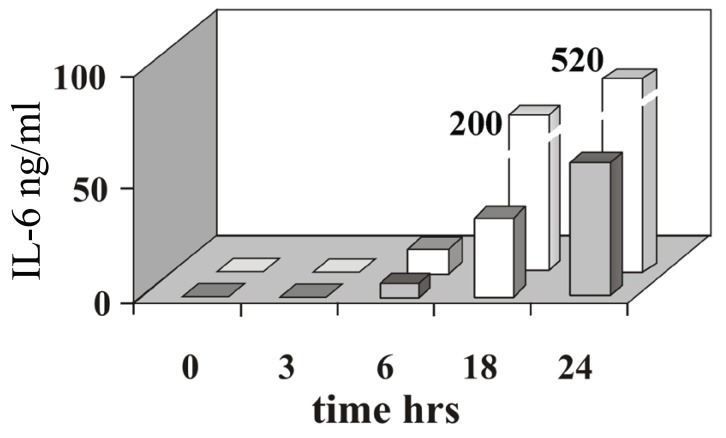
IL-6 induction triggered by TNFα or IL-1β IL-6 (ng/ml) detected by ELISA in the culture medium of cell treated with TNFα (grey) or IL-1β (white), at the time indicated after cytokine induction. Bars representing IL-6 after 18 and 24 hours of incubation with IL-1β are not in scale, the actual values correspond to 200 ng/ml and 520 mg/ml, respectively. The data shown in the figure are representative of three independent experiments.

As detected by ELISA, the time course profile of IL-6 secreted in conditioned medium correlates well with that of mRNA accumulation. IL-6 can be detected after six hours of cell stimulation by either TNFα or IL-1β (Fig. 3). A higher amount of cytokine accumulates in the culture medium of cells treated with IL-1β (up to 200 and 520 ng/ml at 18 and 24 hours, respectively) relative to cell treated with TNFα (40 and 70 ng/ml at 18 and 24 hours, respectively).

### NF-κB activation in the presence of the ROS scavenger NAC

**Figure 4:**
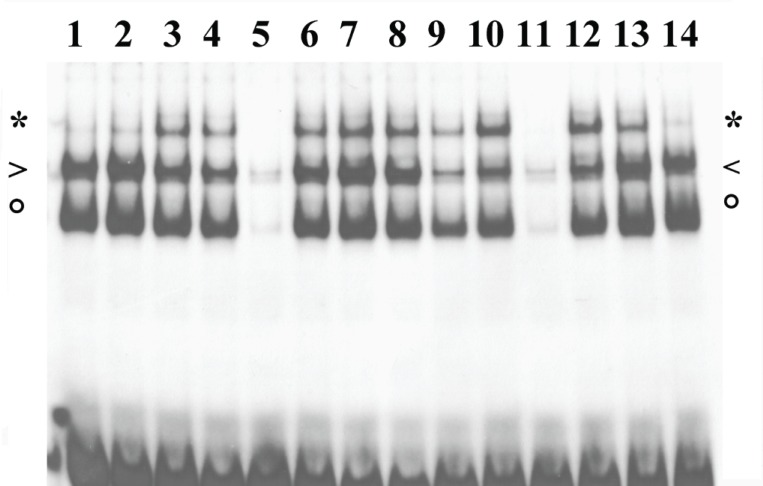
NF-kB activation by TNFα or IL-1β in MG-63 cells in the presence of NAC. MG-63 nuclear extracts were analysed by EMSA in the presence of IL-6κB radioactive probe. Cells were induced with cytokines (3-8 IL-1β, 9-14 TNFα) for 30 min, one hour after addition of NAC (6, 12 15 mM, 7, 13 30 mM, 8,14 50mM). To check binding specificity, 100 fold excess of IL-6κB cold oligonucleotide was added to lanes 5 and 11. Complexes of NF-κB heterodimer, p50 dimer or CBF1 are indicated by asterisk, arrowhead or circle, respectively. The data shown in the figure are representative of three independent experiments.

In many cell types, ROS play an essential role in NF-κB induction by TNFα and IL-1β. Thus we have tested the action of an antioxidant agent, such as NAC, on NF-κB activation triggered by these cytokines on the osteoblast-like MG-63 line. Cells were treated with increasing amounts (15, 30 and 50 mM) of NAC for 1 hour, followed by 30 min induction with either cytokine. NAC pre-treatment raises intracellular GSH levels, thereby protecting the cell from the effects of ROS. The three protein/DNA complexes appearing in EMSA have been previously identified by antibody supershift and/or DNA binding specificity. They contain NF-κB heterodimer, p50 or CBF1 (13, 14). The results (Fig. 4) clearly show the inhibitory effect of the reducing agent on NF-κB activation induced by TNFα, while induction by IL-1β is not impaired by the treatment.

### IL-6 secretion induced by TNFα or IL-1β in the presence of the ROS scavenger NAC

**Figure 5:**
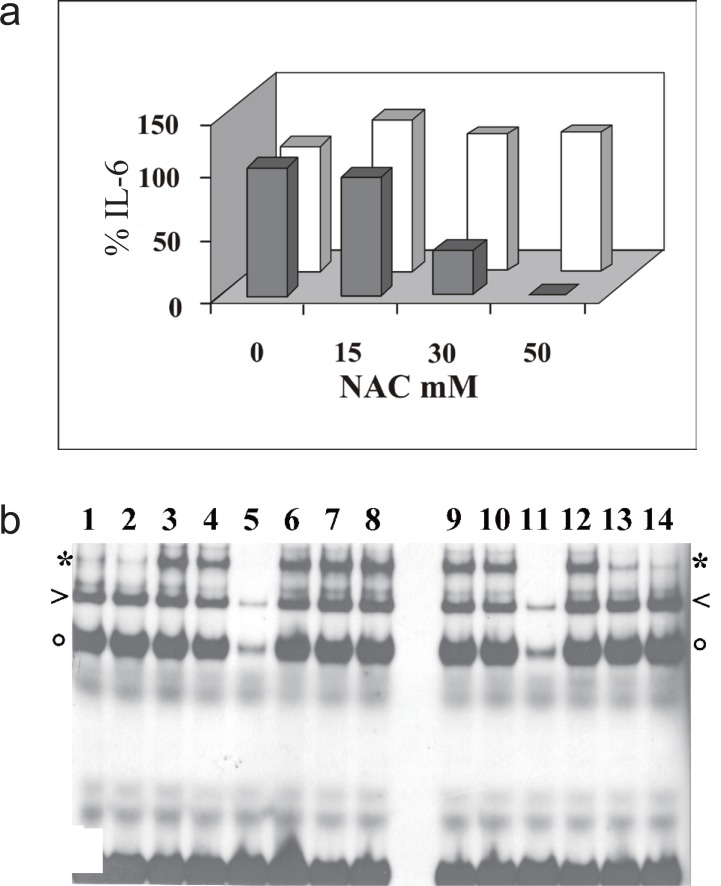
IL-6 induction by TNFα or IL-1β in MG-63 cells in the presence of NAC. a, IL-6 detected by ELISA in the culture medium of cell treated for 6 hours with TNFα (gray) or IL-1β (white), one hour after addition of NAC. Bar height represents the percentage of IL-6 secreted at the indicated concentration of NAC, relative to the value determined in the absence of NAC, taken as 100%; b, EMSA analysis of MG-63 nuclear extracts with the IL-6κB radioactive probe. Cells were treated for 6 hours with IL-1β (3-8) or TNFα (9-14), one hour after addition of NAC (6, 12 15mM, 7, 13 30mM, 8, 14 50mM). To check binding specificity, 100 fold excess of IL-6κB cold oligonucleotide was added to lanes 5 and 11. Complexes of NF-κB heterodimer, p50 dimer or CBF1 are indicated by asterisk, arrowhead or circle, respectively. The data shown in the figure are representative of three independent experiments.

We have then tested the effect of NAC on IL-6 secretion induced by TNFα or IL-1β. In agreement with the central role of NF-κB in transcriptional activation of the IL-6 gene, pre-treatment with NAC of MG-63 cells induced with TNFα strongly reduces the amount of secreted IL-6, while the action of IL-1β remains unaffected (Fig. 5a). We measured IL-6 secreted in conditioned medium after 6 hr of cytokine stimulation, in order to allow accumulation of a detectable amount. At this time too, the NAC-induced decrement of NF-κB binding activity shows a marked dose-dependent profile in cells stimulated with TNFα, while stimulation with IL-1β is not impaired (Fig. 5b).

## DISCUSSION

Disregulated expression of IL-6 has been associated to a variety of inflammatory conditions and inflammation-induced bone loss diseases, such as osteoarthritis, reumatoid arthritis and osteoporosis (15-17). IL-1β and TNFα activate a number of responsive genes in osteoblasts of inflamed joints, among them the IL-6 gene, known to play a complex role in osteoblast and osteoclast differentiation (18).

To shed light on stress cytokines activity in osteoblast-like cells, we have monitored the expression of the IL-6 gene induced by TNFα or IL-1β in the MG-63 cell line derived from a human sarcoma, widely used as a reliable model for osteoblast response to cytokines involved in bone resorbing diseases (19-22). Our data show that both cytokines rapidly induce accumulation of IL-6 mRNA, but IL-1β exhibits a more potent activity than TNFα. This effect is in part due to the higher stability of the IL-6 mRNA induced by IL-1β and the amount of IL-6 secreted in conditioned medium parallels this difference. Thus, despite these cytokines partially share the pathway activating the gene, they clearly elicit a differential regulation on IL-6 expression.

Increasing evidence supports a central role for ROS in IL-1β and TNFα signalling leading to NF-κB activation, even though a number of reports have clearly shown that the involvement and the biochemical source of ROS in these pathways is cell type specific (8, 10, 23, 24). In order to assess if NF-κB induction by these cytokines in MG-63 cells may entail the action of ROS, we have tested the effect of the ROS scavenger NAC. In our experimental system, cell pre-treatment with NAC efficiently counteracts NF-κB activation and IL-6 synthesis triggered by TNFα, whereas stimulation by IL-1β remains unaffected. These results indicate that in MG-63 cells, IL-1β and TNFα signalling pathways do not share a step sensitive to the ROS scavenger. It is unlikely that NAC could perturb the structural stability of these cytokines and/or their receptors. We noted that pre-incubation of the cytokines with NAC does not affects the ability to induce NF-κB activation and IL-6 synthesis (not shown). On the other hand, it has been reported that in intact HeLa cells NAC does not inhibit TNF binding (25). In addition, in KB epidermal cells TNF signalling is resistant to NAC (24). These observations rule out a direct effect of NAC as a thiol reducing agent on the TNFα/ TNFα receptor system even though both proteins, as well as the IL-1 receptors, present disulfide bridges.

This paper presents novel data on the NF-κB-dependent regulation of the IL-6 gene by IL-1β and TNFα in osteoblast-like cells. The activation of NF-κB and induction of IL-6 by TNFα and IL-1β have been reported in different osteoblast-like cell lines (26, 27), the action of these cytokines, however, has not been comparatively investigated. We have shown their differential sensitivity to NAC, suggesting the involvement of ROS production only in TNFα signalling. This difference may be important in the control of IL-6 expression for therapeutic purposes. It is known that osteoblasts in inflamed joints upon IL-1β and TNFα stimulation synthesize, among other cytokines, IL-6, which is known to play a central role in differentiation of osteoclast, responsible for bone resorption. Osteoblasts are relevant components in the complex cell population including chondrocytes, bone marrow stromal cells, synovial fibroblasts and blood monocytes involved in inflammatory bone resorbing diseases. Thus, unravelling the regulation of NF-κB pathway and IL-6 synthesis in this cell type is important for developing new strategies for treatments of bone diseases.
